# Evaluation of the color stability of three maxillofacial silicone materials after exposure to beverages: An in vitro study^☆^

**DOI:** 10.1016/j.heliyon.2024.e25529

**Published:** 2024-01-30

**Authors:** Anshul Chugh, Mariko Hattori, Cheewin Towithelertkul, Yuka I. Sumita, Noriyuki Wakabayashi

**Affiliations:** a Department of Advanced Prosthodontics, Graduate School of Medical and Dental Sciences, Tokyo Medical and Dental University, Tokyo, Japan; b Department of Prosthodontics, Crown and Bridge, Postgraduate Institute of Dental Sciences, Haryana, India; c Department of Prosthodontics, Maxillofacial prosthetics services, Mahidol University, Bangkok, Thailand; d Division of General Dentistry 4, The Nippon Dental University Hospital, Tokyo, Japan; e Tokyo Medical and Dental University, Tokyo, Japan

## Abstract

**Purpose:**

Oral cancer often requires treatments like surgical intervention, involving surgical resection of lips and other facial parts. For those patients, prosthetic rehabilitation following surgery promotes social reintegration. This study explores the color stability of various commercially available silicone materials for facial prostheses when exposed to everyday beverages like coffee and tea. The hypothesis is that these beverages can induce color changes in silicones, simulating conditions of daily use.

**Material and methods:**

A total of 90 specimens were fabricated. There were 30 specimens deefor each of 3 nonpigmented silicone elastomers: Silfy, A-2186-F and VST-50 and were immersed in tea or coffee at drinking temperature and evaluated for color changes at time points of 0 h, 1 h, and 6 h. The color was measured with a colorimeter that used the CIE L*a*b* system and the color change ΔΕ was calculated to quantify the color change. Statistical analysis was performed by using the Kruskal-Wallis test, the Mann-Whitney *U* test, and the Wilcoxon signed-rank Test. The threshold for statistical significance was P < .05.

**Results:**

Color change was observed for each of the silicones exposed to the beverages (P < .05). The value of ΔE was significantly higher for A-2186-F compared with VST-50 and Silfy (P < .05 in all beverages at 6 h). The color of the elastomers progressively changed while immersed in the beverages over 6 h. The change in color was significantly greater for coffee compared with tea (P < .05).

**Conclusions:**

The color stability of nonpigmented silicone elastomers is inherently low, which contributes to the overall color change of silicone prostheses when they are exposed to beverages that contain pigments. The elastomer A-2186-F had greatest values of ΔE among the materials tested. Color changes of silicone prostheses can be lessened by selecting materials with consideration of color stability to improve their longevity and extend their period of use.

## 1 Introduction

Head and neck cancer stands as the seventh most prevalent cancer worldwide, with oral cancer emerging as its most common form. Focusing on malignancies impacting the lips, oral cavity, and oropharynx, oral cancer is a substantial global health concern, securing the 13th position in cancer prevalence [1]. Dominated by squamous cell carcinoma and basal cell carcinoma, oral cancer presents a range of complex challenges. These encompass the risk of disfigurement and the potential to impair essential physiological functions. Beyond the evident physical effects, head and neck cancer triggers pathophysiological changes that negatively impact diverse bodily functions, leading to nutritional deficiencies and social isolation. Consequently, these factors intricately shape an individual's overall well-being [[2], [3], [4]]. Surgical intervention remains the fundamental approach in managing oral cancer, its specifics contingent upon the nature and extent of the tumor. However, certain clinical scenarios may make surgical reconstruction unfeasible, leading to alternative solutions like the implementation of silicone lip prostheses. Particularly in cases involving midfacial defects that encompass the oral cavity, lips, eyes, or nasal regions, midfacial prosthesis along with silicone lip prostheses serve as a practical option when surgical reconstruction is not viable. Nevertheless, their use is not without challenges. Complications such as salivary influx, prosthetic seal breakage, and margin show-through during articulatory activities pose difficulties, potentially resulting in fluid spillage during dietary intake. The integration of combination prostheses, such as an obturator with a lip prosthesis or a midfacial prosthesis involving nasal or eye components, introduces additional considerations, as fluid seepage can impact their properties. Recognizing and addressing these complexities are crucial for optimizing the efficacy and overall patient outcomes associated with silicone lip prosthesis in the context of lip cancer treatment [5,6].

The search for the ideal material for maxillofacial prosthetics has continued from their beginning up to the present day. The earliest materials used were wood, ivory, waxes, and metals. In 1945, Clarke described techniques for fabricating prosthesis from latex rubber, glycerin gelatin, and electroplated metals [7]. Lewis et al. [8], classified the ideal properties of these materials into 3 categories: (1) processing characteristics, such as viscosity and working time; (2) mechanical or performance characteristics, such as tear strength, hardness, and dimensional stability; and (3) patient-centered properties. The medical-grade silicones are either room-temperature or heat vulcanized. Silicone elastomers are supplied in a 2-part system and are usually mixed in a 10:1 ratio by weight or volume. Silicone elastomers are classified according to their application: Class 1 is implant grade used for breast augmentation, Class II is medical grade used for maxillofacial prosthesis, and Class III is clean grade used in food coverage and packaging, and Class IV is industrial grade used for industrial applications [9,10].

Discoloration of materials reflects the surrounding environment. Solar radiation, moisture, temperature, airborne pollutants, and routine cleaning can cause color changes in maxillofacial silicone prosthesis. Hatamleh and Watts [ [11]], stated that the color stability of non-pigmented silicone elastomers is inherently low, which contributes to overall color changes over time. Silicone cleaning solutions on their own did not cause perceivable color changes in specimens. Griniari and coworkers [ [12]], also reported that color changes for the materials tested were within the limits of clinical acceptability after all aging procedures. Immersion in distilled water presented the best color stability.

Elastomers are more color stable than other materials used in maxillofacial prosthesis. Many authors have investigated the color stability of maxillofacial silicones under various conditions, such as weathering, artificial aging, and use of disinfectants [13,14]. A study by Sethi et al. [15], in 2015 found that materials used for mold fabrication and separating media have a clear impact on the color of silicone. Haug and Farah [16], also reported that changes in optical properties and color occur in both colored and non-colored silicone specimens subjected to weathering conditions [17]. In addition to weathering and aging, cosmetics, food, and beverages can also affect color and require attention to improved materials since facial prostheses are close to the oral area [8].

Color stability in facial prostheses is measured using devices like colorimeter and spectrophotometers. Determining which color changes are both noticeable and considered acceptable poses a challenge. In this context, "noticeable" refers to changes that can be seen, while "acceptable" refers to the level that is considered satisfactory from a clinical perspective. However, which color changes of facial prostheses are visually perceptible, and which are clinically acceptable is still unclear as different values for perceptible thresholds and acceptable thresholds have been reported [18,19]. Although the color stability of facial prosthesis in solar radiation, moisture, temperature variations, airborne pollutants, and routine cleaning are often discussed in literatures [20,21], there has been no report on the color change of silicone for the when exposed to everyday beverages. The aim of this study was to compare 3 non-pigmented silicone elastomers and color changes under conditions simulating the regular consumption of hot beverage by a patient wearing a silicone lip prosthesis. The null hypothesis was that regularly consumed hot beverages will not change the color of silicone elastomers.

## 2 Material and methods

A total of 120 specimens were made by using 3 Class II commercially available silicone materials. Of these 30 were rejected because of voids and defects and 90 specimens were selected for use after inspection confirmed no voids or defects. No color was added to the silicones and thus they remained nonpigmented, as the 3 types of silicone elastomers were A-2186-F (Platinum RTV Silicone Elastomer A-2186-F; Factor II), VST-50 (Platinum Silicone Elastomer VST-50; Factor II), and Silfy (Gc Silfy; GC).

The 90 specimens were made of size (20 mm of length, width and 2 mm thickness) to simulate the thickness of a maxillofacial prosthesis. Then, silicone is hand-mixed in a ratio of 10:1 base to catalyst for 30 s and then vacuum-mixed for 60 s. After being mixed, the silicone was loaded into syringe to reduce voids and the materials were loaded equally into preformed molds. The room-temperature silicones were then allowed to vulcanize at room temperature for 24 h and the other silicones were kept in a dry oven at 100 °C for 2 h. The specimens were randomly allocated into 18 groups (Table 1). Each group had silicone immersed in beverages for 1 h and 6 h (n = 5).

**Table 1:** Materials used in the study.

Material		Brand name	Manufacturer
Silicone Elastomer		Gc Silfy	GC, Tokyo, Japan
		Platinum Silicone Elastomer VST-50	Factor II, Inc., Lakeside AZ, USA
		Platinum RTV Silicone Elastomer A-2186-F	Factor II, Inc., Lakeside AZ, USA

Material Beverages	Brand name	Processing method	Manufacturer

Green tea	Happy Belly Ito En Domestic Green Tea	Immersing the tea bag in hot water for 3 min	Ito En, Tokyo Japan.
Indian tea	Brooke Bond Red Label Tea	Immersing the tea bag in hot water for 3 min	Brooke Bond Red Label, Hindustan Unilever Limited, Mumbai, India.
English tea	Twinings, Earl Grey	Immersing the tea bag in hot water for 3 min	Twinings and Company, London, UK.
Indian coffee	Nescafe Classic	Hot water was passed slowly poured onto roasted ground coffee contained in a filter	Nestle India Ltd., Punjab, India.
Japanese coffee	Tokyo Roast	Hot water was passed slowly poured onto roasted ground coffee contained in a filter	Ueshima coffee company, Hyogo, Japan.
Turkish coffee	Hafiz Mustafa Turkish Coffee	Hot water was passed slowly poured onto roasted ground coffee contained in a filter	Hafiz Mustafa, Istanbul, Turkey.

After vulcanization the silicones were immersed in beverages. Beverages regularly consumed in various countries were used: Green tea **(**Happy Belly Ito En Domestic Green Tea; Ito En, Tokyo Japan), Indian tea (Brooke Bond Red Label Tea; Brooke Bond Red Label, Mumbai, India), English tea (Twinings Earl Grey; Twining and Company, London, UK), and Japanese coffee (Tokyo Roast; Ueshima Coffee Company, Hyogo, Japan), Turkish coffee (Hafiz Mustafa Turkish Coffee; Hafiz Mustafa, Istanbul, Turkey) and Indian coffee (Nescafe Classic; Nestle India Ltd., Punjab, India). The tea and coffee were prepared according to the manufacturer's instructions and then the specimens were immersed in the beverages in containers away from sunlight and other weathering conditions that would affect the color of the non-pigmented silicone (Table 1).

The specimens were left in the beverages at 37 °C in an oven simulate the drinking temperature in the oral cavity. Then, the specimens were evaluated by using colorimeter (Color Reader CR13; Konica, Tokyo, Japan) at different time intervals, to simulate an average person's beverage consumption over approximately a day. The specimens in each beverage were evaluated for color changes at time points before and after immersion.

Color was described by using the Commission International d’Exchange (CIE) L* a* b* system. The measurements were performed with a colorimeter according to CIELab coordinates. To determine the color of the test specimens, the colorimeter was calibrated according to the manufacturer's instructions by using the white calibration standard supplied. Measurement was performed at 3 different locations for each specimen. Mean levels of L*, a*, and b* were automatically calculated by the colorimeter and recorded in the CIELab color system, a color order space with coordinates for lightness: white-black (L*), red-green (a*), and yellow-blue (b*). An initial measurement of CIELab color values was first performed at 0 h before immersion, and then the measurement was repeated in the same manner after immersion of the silicone for 1 h and 6 h.

**Fig. 1:**
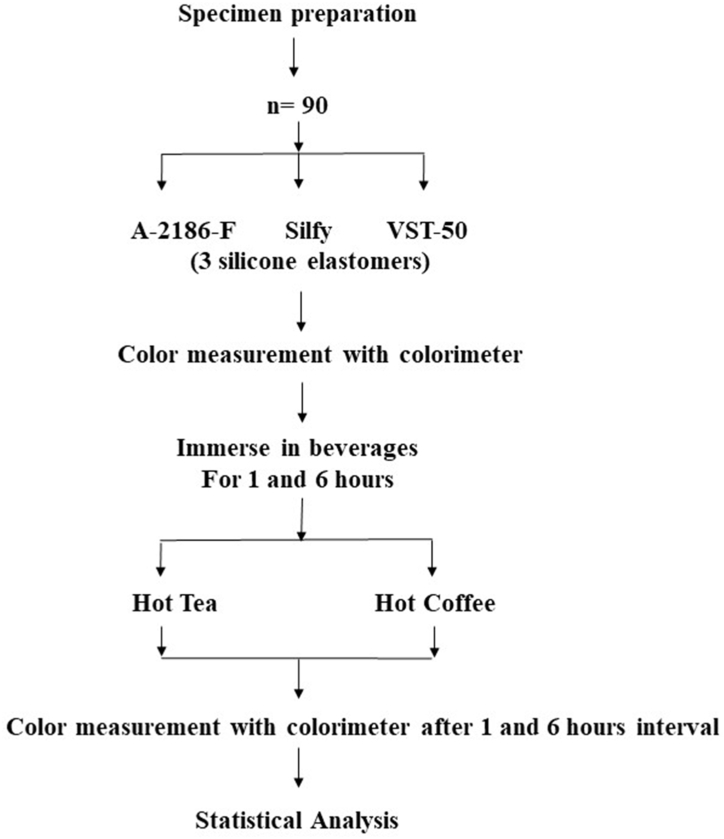
Schematic presentation of Materials and methods.

The color change ΔE and other variables (L*, a*, and b*) of these silicones used in various maxillofacial prostheses was compared between the groups with respect to the type of beverage and duration of immersion. The color change ΔE was calculated by using the following equation: ΔE=([ΔL∗]^2^ + [ Δa∗]^2^ + [Δb∗]^2^) ^½.^ Here, ΔL∗ is the difference in L∗, Δa∗ is the difference in a∗, and Δb∗ is the difference in a∗ between different time points. Collected data was compiled in a spread sheet (MS Office Excel, Redmond, WA, USA) and was subjected to statistical analysis by using SPSS ver. 28.0 (IBM, IL, USA). Descriptive statistics for color change ΔE with respect to L, a, and b were calculated for all samples and for the different groups (Fig. 1).

Statistical analyses were conducted employing the Kruskal-Wallis test to assess comparisons among three distinct silicone elastomers in different beverages. Pairwise comparison was done with the Mann-Whitney *U* test with Bonferroni correction and the Wilcoxon signed-rank test for the intragroup comparison. For the statistical tests, P < .05 considered statistically significant, with α error kept at 5 % and β error kept at 20 %, thus giving statistical power of 80 %.

## 3 Results

Color changes were observed and compared among the groups. Based on the 50:50 % acceptability threshold (ΔE = 3.0), the interpretation of the data of color changes in this study was done [19,22]. Intergroup and intragroup comparisons of ΔE were done at time points of 1 h and 6 h for Silfy, VST-50, and A-2186-F as shown in the graph and tables.

The Kruskal-Wallis test was used for intergroup comparison between Silfy, VST-50, and A-2186-F. Results showed significant differences between the groups for the different teas and coffees. For the teas, differences in ΔE among the silicones were observed, with VST-50 showing significantly higher ΔE in English tea (median ΔE = 2.385372; ꭓ^2^ = 11.80, P = .004) and in Indian tea (median ΔE = 2.161018; ꭓ^2^ = 6.080, P = .048) at 1 h. At the 6-hourtime point, A-2186-F showed significantly higher values of ΔE in comparison with Silfy and VST-50 for all the beverages tested (all P < .05; Table 2). Significant differences in ΔE values were found between the groups for the different coffees (Japanese, Indian, and Turkish). At 1 h, A-2186-F showed significantly higher values of ΔE in Japanese coffee (median ΔE = 3.189044) and Indian coffee (median ΔE = 7.783315). At 6 h, A-2186-F showed significantly higher values in Japanese coffee (median ΔE = 6.886218) and Indian coffee (median ΔE = 12.64041). VST-50 showed higher values of ΔE in Turkish coffee at 1 h (median ΔE = 7.66942) and 6 h (median ΔE = 10.30097) (Table 2).

**Table 2:** Median scores for ΔE values for different silicones immersed in different beverages at different time points.

At 1 h						
Silicone elastomers	Green tea	English tea	Indian tea	Japanese coffee	Turkish coffee	Indian coffee
A-2186-F	2.184033	1.240967	2.949576	3.189044	4.047221	7.783315
Silfy	1.870829	0.640312	1.004988	1.964688	5.615158	2.083267
VST-50	2.385372	2.161018	0.943398	2.304344	7.66942	2.302173
ꭓ^2^	1.280	11.180	6.080	6.620	9.060	9.620
P-value*	.527	.004*	.048*	.037*	.011*	.008*
**At 6 h**						
A-2186-F	3.920459	5.813777	7.937884	6.886218	7.072482	12.64041
Silfy	1.982423	2.161018	4.545327	4.455334	6.354526	3.911521
VST-50	1.946792	2.609598	3.052868	3.706751	10.30097	7.256032
ꭓ^2^	6.860	11.200	8.880	10.500	7.280	9.920
P-value*	.032	.004*	.012*	.005*	.026*	.007*

Pairwise comparisons by the Mann-Whitney *U* test between Silfy and A-2186-F and between A-2186-F and VST-50. A significant difference was observed for ΔE between Silfy and VST-50 in English tea (z = −2.619, P = .009) and in Japanese coffee (z = −2.611, P = .009) at 6 h, while no significant difference was seen at 1 h.

Comparison between A2186-F and Silfy, statistically significance in color change was seen in English tea and Indian coffee at 1 h and 6 h (z = −2.619, P = .009). There was non-significant difference for ΔE in Green tea, Indian tea, Japanese coffee, and Turkish coffee (Table 3).

**Table 3:** Pairwise comparison of silicone elastomer in different beverages at different time points.

Time (1 h)	Mann-Whitney U (P < .05)	Green tea	English tea	Indian tea	Japanese coffee	Turkish coffee	Indian coffee
A-2186-F Silfy	Z	−1.358	−2.611	−1.776	−1.984	−1.358	−2.611
	P value	.175	.009*	.076	.047	.175	.009*
A-2186-F VST-50	Z	−0.313	−2.611	−2.402	−2.402	−2.611	−2.611
	P value	.754	.009*	.016*	.016*	.009*	.009*
Silfy VST-50	Z	−0.313	−1.984	−0.104	−0.313	−2.193	−.731
	P value	.754	.047	.917	.754	.028	.465

Time (6 h)	Mann-Whitney U	Green tea	English tea	Indian tea	Japanese coffee	Turkish coffee	Indian coffee

A-2186-F Silfy	Z	−1.984	−2.619	−1.776	−1.567	−0.522	−2.611
	P value	.047	.009*	.076	.117	.602	.009*
A-2186-F VST-50	Z	−2.402	−1.984	−2.402	−2.611	−2.611	−2.402
	P value	.016*	.047	.016*	.009*	.009*	.016*
Silfy VST-50	Z	−0.522	−2.619	−2.193	−2.611	−1.984	−1.567
	P value	.602	.009*	.028	.009*	.047	.117

* Shows the significant difference for pairwise comparison.

Comparison between A-2186-F and VST-50 showed statistically significant change in color at 1 and 6 h in Indian tea (z = −2.402, P = .016), Japanese coffee, (z = −2.402, P = .016), Turkish coffee and Indian coffee (z = −2.611, P = .009) while change was seen in English tea at 1 h (z = −2.611, P = .009) and Green tea at 6 h (z = −2.402, P = .016). (Table 3).

Intragroup comparisons using the Wilcoxon signed-rank test was done at 1 h and 6 h for Silfy, VST-50, and A-2186-F with statistical significance set at P < .05. In the Green tea, there was no significant change in color between 1 h and 6 h for A-2186-F (P = .80), Silfy (P = .893), and VST-50 (P = .686). In Turkish coffee, there was no significant change in color between 1 h and 6 h for A-2186-F (P = .225), but significant changes were observed for Silfy and VST-50 (P = .043). In English tea, Indian tea, Japanese coffee, and Indian coffee significant changes in color were observed between 1 h and 6 h for A-2186-F, Silfy, and VST-50 (all P < .05) (Table 4).

**Table 4:** Intragroup comparison of 3 different silicone elastomers in different beverages at 1 h and 6 h.

Silicone elastomer	Wilcoxon Signed-Rank Test (P < .05)	GT1 – GT6	ET1 – ET6	IT1 – IT6	JC1 – JC6	TC1 – TC6	IC1 – IC6
A-2186-F	Z	−1.753	−2.023	−2.023	−2.023	−1.214	−2.023
	P value	.080	.043*	.043*	.043*	.225	.043*
Silfy	Z	−0.135	−2.023	−2.023	−2.023	−2.023	−2.023
	P value	.893	.043*	.043*	.043*	.043*	.043*
VST-50	Z	−0.405	−2.023	−2.023	−2.023	−2.023	−2.023
	P value	.686	.043*	.043*	.043*	.043*	.043*

GT- Green tea, ET- English tea; IT- Indian tea; JC- Japanese coffee; TC- Turkish coffee; IC- Indian coffee. * Shows the significant difference.

Among the silicone elastomers, the color change of VST-50 and Silfy among Green tea and English tea at 6 h were below the acceptability threshold of ΔE = 3.0. The color change of A-2186-F among all beverages, whereas VST-50 and Silfy among Indian tea and all coffees after 6 h, were above the accessibility threshold of ΔE = 3 (Table 2).

**Fig. 2:**
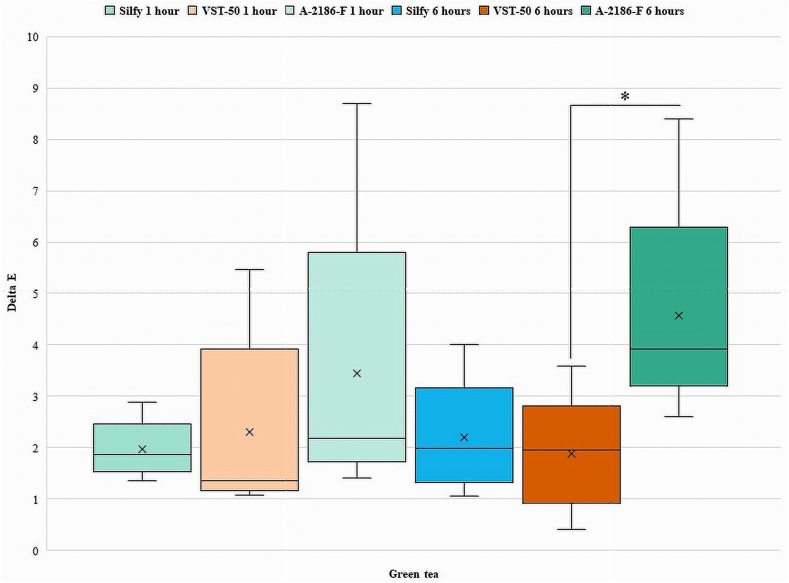
Comparison of ΔE of silicone elastomers in Green tea at 1 h and 6 h *Statistically significant (P < .05). (For interpretation of the references to color in this figure legend, the reader is referred to the Web version of this article.)

**Fig. 3:**
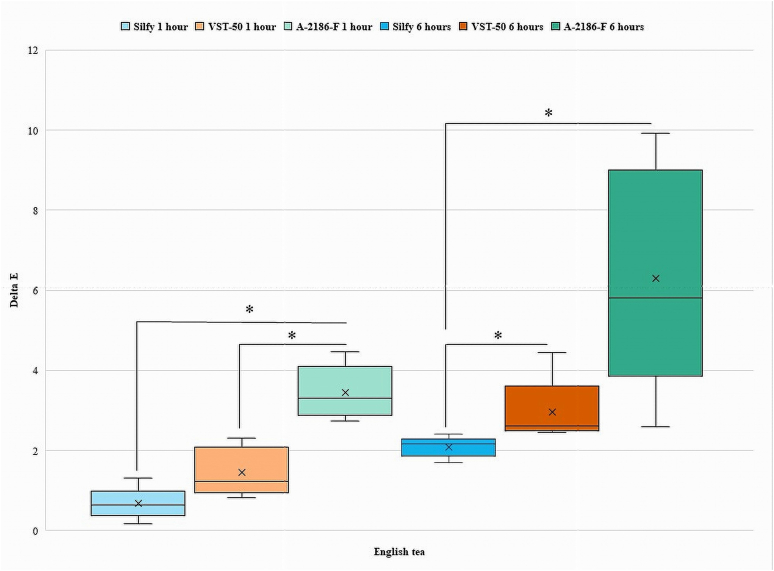
Comparison of ΔE of silicone elastomers in English tea for 1 h and 6 h *Statistically significant (P < .05).

**Fig. 4:**
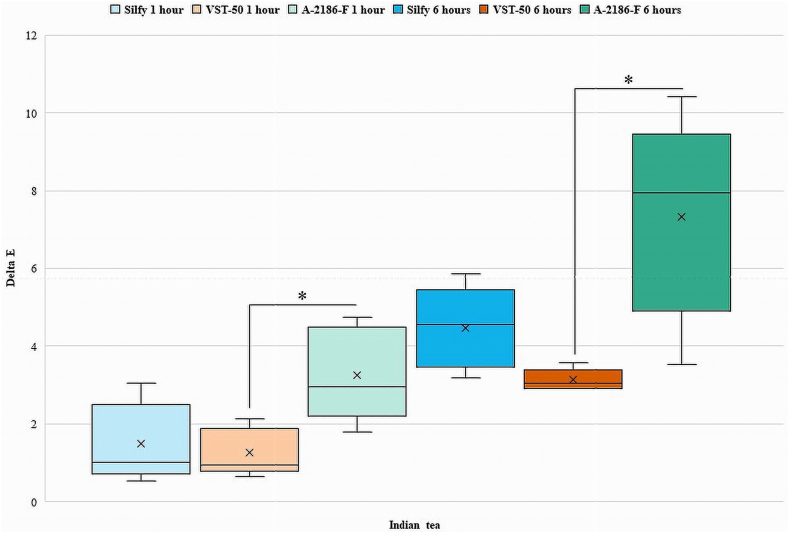
Intergroup comparison of ΔE of silicone elastomers in Indian tea for 1 h and 6 h *Statistically significant (P < .05).

**Fig. 5:**
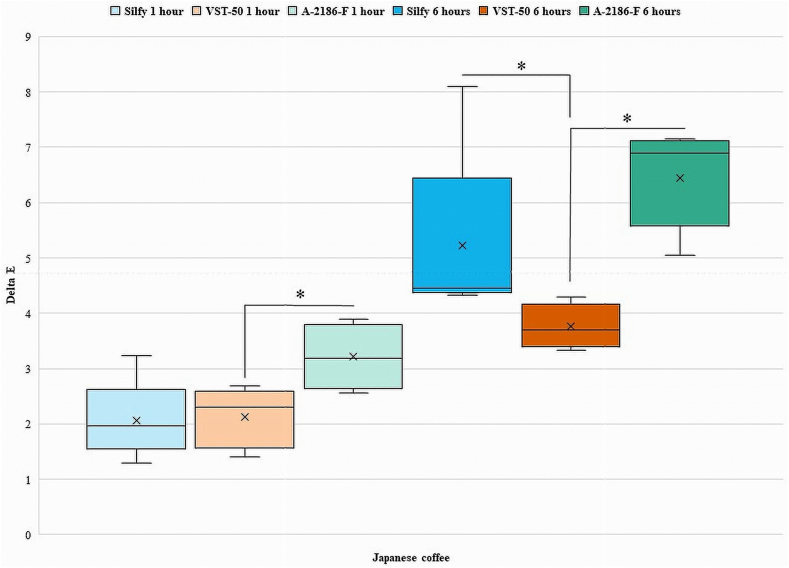
Intergroup comparison of ΔE of silicone elastomers in Japanese coffee for 1 h and 6 h *Statistically significant (P < .05).

**Fig. 6:**
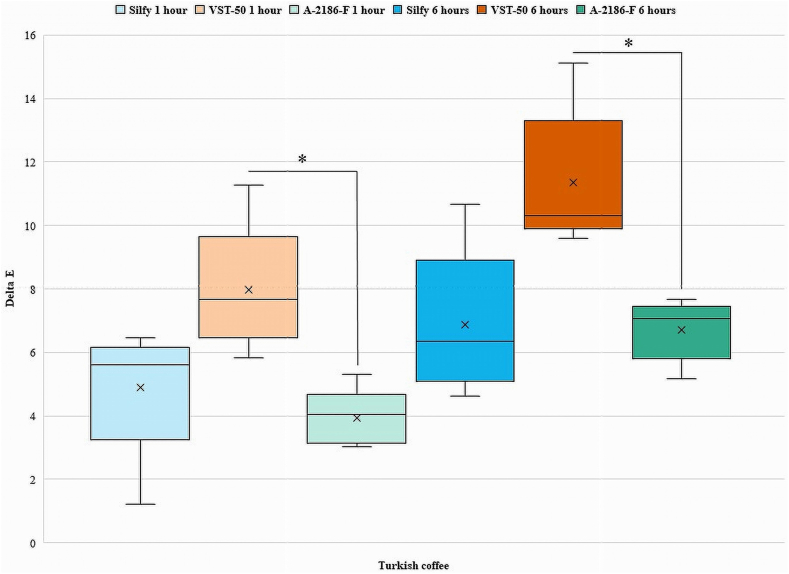
Intergroup comparison ΔE of silicone elastomers in Turkish coffee for 1 h and 6 h *Statistically significant (P < .05).

**Fig. 7:**
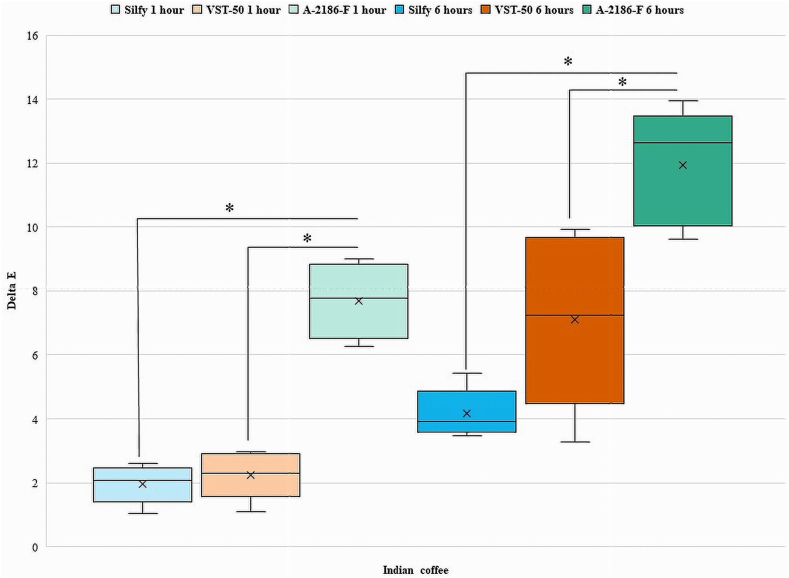
Intergroup comparison of ΔE of silicone elastomers in Indian coffee for 1 h and 6 h *Statistically significant (P < .05).

Fig. 2, Fig. 3, Fig. 4, Fig. 5, Fig. 6, Fig. 7 show the ΔE values among different silicones at different time points. The ΔE values showed a progressive change in color from 1 h to 6 h. With time the ΔE values have increased showing that color changes with time of consumption of hot beverages. The beverages had less of an effect on Silfy compared with the other 2 silicones (A-2186-F and VST-50). Among beverages coffee has more effect in color change than tea.

## 4 Discussion

It is said that one of the most important factors for patients to accept a facial prosthesis is how well its color stays. This is because facial prosthetics often need replacement, mainly due to changes in appearance and color over time [22].

The colors of different silicones changed with the exposure to the beverages and thus the null hypothesis was rejected. The results indicate that the ΔE value was consistently higher than zero for all samples, signifying color alteration. Existing literature suggests that this color change may arise from both intrinsic and extrinsic factors [23]. Intrinsic factors involve inherent material discoloration with matrix changes, while extrinsic factors, such as the absorption and adsorption of staining agents, can also lead to discoloration [24,25]. Color changes were observed across all groups, with A-2186-F showing significant alterations (P < .05). Notably, beverages like coffee and tea had an impact on the color changes in these non-pigmented silicones. Paravina et al. [19] found that color changes of ΔΕ = 1.1 and more were perceptible and ΔΕ = 3 and less were acceptable for light skin colored silicone elastomers. On the other hand, it is important to interpret the data obtained in color studies based on the 50:50 % acceptability threshold (ΔE = 3) [22,26]. Based on above studies, the present study accepted that, the acceptability threshold of ΔΕ = 3. Therefore, both statistical differences between the groups and acceptability thresholds were taken into consideration while interpreting the data in this study.

**Fig. 8:**
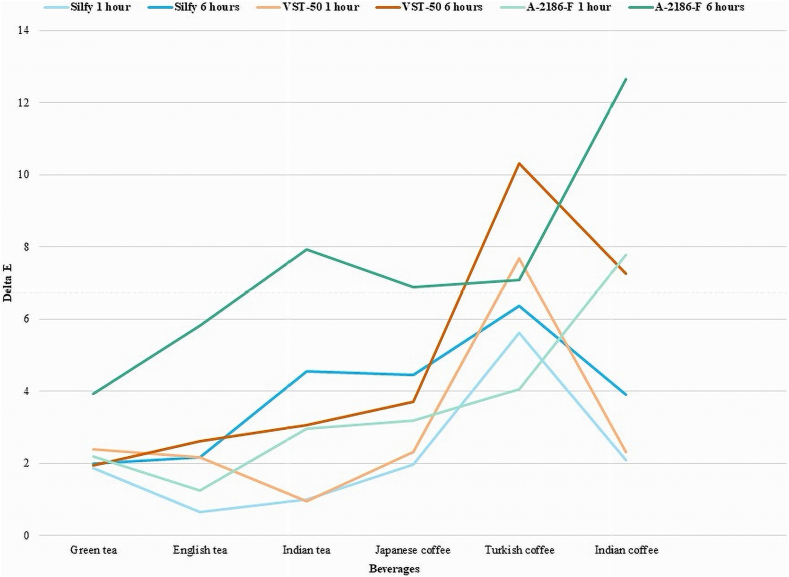
Median score of ΔE of 3 silicone elastomers in hot beverages.

Among the beverages tested, Indian coffee induced the most significant change, followed by Turkish coffee, while Green tea had the least impact, indicating that coffee had a more pronounced effect on discoloration than teas. A continuous and significant color change was observed over time, from 0 to 6 h, as illustrated in Fig. 8, depicting the median values of ΔE for silicone elastomers and the trend of color change over time. Early uptake of pigment compounds in beverages may have contributed to subsequent changes observed after 6 h. Challenges such as salivary influx, prosthetic seal breakage, and margin show-through during articulatory activities present difficulties that may lead to fluid spillage during dietary intake, hypothesized as a reason for discoloration [5,6].

The observed changes in color of the silicones after exposure to beverages were consistent with results reported by Eleni et al. [27], who assessed 3 different types of polydimethylsiloxanes after immersion in simulated body fluid and sweat at 37 °C and showed that there were significant color changes in all materials afterward. Also, in 2 different experiments concerning pigmentation of maxillofacial silicones under different aging conditions, Polyzois [28], observed color differences (ΔE) ranging from 2 to 3.5 units and 2.13–3.98 units. According to Polyzois [29], the duration of the exposure and the type of silicone were significant factors affecting color stability. According to Goiato et al. [30], it was observed that colorless silicone underwent alterations when exposed to various substances commonly used for cleaning prostheses, as well as extreme weather conditions such as ultraviolet B radiation and cycles of water condensation in an accelerated aging chamber. These findings align with other studies indicating that colorless silicone exhibited chromatic alterations irrespective of the presence of added pigments and opacifiers. Both unpigmented and pigmented specimens demonstrated changes in color, suggesting that the elastomer itself played a role in contributing to the observed color alterations in pigmented specimens [31,32].

Griniari et al. [33], showed no structural changes after immersion in solutions and photoaging, for all materials evaluated. No statistically significant differences in ΔΕ among the tested groups were found in that study. This conflicts with the present results, and this can likely be explained by their use of one type of silicone.

The color of an object, as perceived by the human eye, is determined by the wavelength of light reflected from its surface [26].The human eye can detect a difference in hue (red, yellow, green, blue, and so on), chroma (saturation), and lightness to the same degree. The color change ΔΕ of the non-pigmented specimens of all the tested silicone elastomers was greater than the acceptable value (ΔE = 3), except in Green tea and English tea for Silfy and VST-50 at 6 h (ΔE = 1.982423, ΔE = 2.161018, VST-50 ΔE = 1.946792 and ΔE = 2.609598, in Green and English tea respectively). The elastomer A-2186-F showed the greatest change, and Silfy showed the least.

Though there are some limitations of this study due to the use of non-pigmented silicone, it can be hypothesized that the addition of pigments may provide more color stability to the maxillofacial silicones. The perceptibility threshold could also be added to in addition to accessibility threshold. The period of exposure should be prolonged for evaluating change in the color. Also, this is in vitro study, but evaluation of maxillofacial elastomer in direct contact with human skin over a longer time would involve exposure to beverages and other outdoor or weather conditions in daily life. This would enable the real effect to be assessed.

In this study, hot beverages were used; future more research can be done with cold beverages and other food items, using other ways of calculating ΔE such as CIEDE2000 and comparing two ways of calculations.

## 5 Conclusions

Based on the findings in this vitro study, the inherent color instability of non-pigmented silicone elastomers was observed, which contributed to the overall color change of these silicone prostheses after exposure exposed to the tested beverages. Among the 3 materials tested, A-2186-F showed the greatest values of ΔE in most of the hot beverages used. There was continuous change in color with time. All the hot beverages produced color change in the silicones, but the change in color was larger for coffee than for tea. All the silicones tested showed color changes, but they were smallest for Silfy, which demonstrated the best color stability overall. Materials for maxillofacial prostheses should be selected with consideration of color stability in order to improve the longevity of the prostheses and extend their period of use.

## Data availability statement

The data will be available on request.

## CRediT authorship contribution statement

**Anshul Chugh:** Writing – original draft, Validation, Software, Data curation. **Mariko Hattori:** Writing – review & editing, Supervision. **Cheewin Towithelertkul:** Visualization, Investigation. **Yuka I. Sumita:** Methodology, Conceptualization. **Noriyuki Wakabayashi:** Supervision.

## Declaration of competing interest

The authors declare that they have no known competing financial interests or personal relationships that could have appeared to influence the work reported in this paper.

## References

[1] The Global Status Report on Oral Health 2022. https://www.who.int/team/noncommunicable-diseases/global-status-report-on-oral-health-2022/.

[2] Kerawala C., Roques T., Jeannon J.P., Bisase B. (2016). Oral cavity and lip cancer: United Kingdom National multidisciplinary guidelines. J. Laryngol. Otol..

[3] Zeno H.A., Sternberger S.S., Tuminelli F.J., Billotte M., Kurtz K.S. (2013). Combination lower lip prosthesis retained by an intraoral component. J. Prosthodont..

[4] Sayed S.I., Elmiyeh B., Rhys-Evans P., Syrigos K.N., Nutting C.M., Harrington K.J. (2009). Quality of life and outcomes research in head and neck cancer: a review of the state of the discipline and likely future directions. Cancer Treat Rev..

[5] Alqarni H., Montgomery P., Aponte-Wesson R., Won A.M., Hofstede T.M., Chambers M.S. (2022). Combined rehabilitation of a lower lip defect after resection of floor of mouth cancer: a clinical report. J. Prosthet. Dent.

[6] Singh N., Singh S.V., Arya D. (2020). Mechanically retained functional prosthetic rehabilitation of partial lip necrosis: a rare clinical report. J. Indian Prosthodont. Soc..

[7] Clarke C.D. (1946). Facial and body prosthesis. Bull. Sch. Med. Univ. Md..

[8] Lewis D.H., Castleberry D.J. (1980). An assessment of recent advances in external maxillofacial materials. J. Prosthet. Dent.

[9] Shen C., Rawls H.R., Esquivel-Upshaw J.F. (2021).

[10] (1975). Maxillofacial prosthetic materials. Council on dental materials and devices. J Am Dent Assoc.

[11] Hatamleh M.M., Watts D.C. (2010). Effect of extraoral aging conditions on color stability of maxillofacial silicone elastomer. J. Prosthodont..

[12] Griniari P., Polyzois G.L., Papadopoulos T., Hatamleh M.M., Watts D.C., Polyzois G.L. (2000). Color and structural changes of a maxillofacial elastomer: the effects of accelerated photoaging, disinfection and type of pigments. J. Prosthet. Dent.

[13] Craig R.G., Koran A., yu R., Spencer J. (1978). Color stability of elastomers for maxillofacial appliances. J. Dent. Res..

[14] Al-Harbi F.A., Ayad N.M., Saber M.A., Arrejaie A.S., Morgano S.M. (2015). Mechanical behavior and color change of facial prosthetic elastomers after outdoor weathering in a hot and humid climate. J. Prosthet. Dent.

[15] Sethi T., Kheur M., Coward T., Patel N. (2015). Change in color of a maxillofacial prosthetic silicone elastomer, following investment in molds of different materials. J. Indian Prosthodont. Soc..

[16] Haug S.P., Keith Moore B., Andres C.J. (1999). Color stability and colorant effect on maxillofacial elastomers. Part I1: weathering effect on physical properties. J. Prosthet. Dent.

[17] Farah A., Sherriff M., Coward T. (2018). Color stability of nonpigmented and pigmented maxillofacial silicone elastomer exposed to 3 different environments. J. Prosthet. Dent.

[18] Leow M.E.L., Ow R.K.K., Lee M.H., Huak C.Y., Pho R.W.H. (2006). Assessment of colour differences in silicone hand and digit prostheses: perceptible and acceptable thresholds for fair and dark skin shades. Prosthet. Orthot. Int..

[19] Paravina R.D., Majkic G., Del Mar Perez M., Kiat-Amnuay S. (2009). Color difference thresholds of maxillofacial skin replications. J. Prosthodont..

[20] Huber H., Studer S.P. (2002 Feb 1). Materials and techniques in maxillofacial prosthodontic rehabilitation. Oral Maxillofac. Surg. Clin..

[21] Tran N.H., Scarbecz M., Gary J.J. (2004 May 1). In vitro evaluation of color change in maxillofacial elastomer through the use of an ultraviolet light absorber and a hindered amine light stabilizer. J. Prosthet. Dent.

[22] Kiat-amnuay S., Cevik P., Kurtoglu C. (2023). Effect of thixotropic agent on the color stability of platinum-based silicone maxillofacial elastomers after artificial aging. Materials.

[23] Villalta P, Lu H, Okte Z, Garcia-Godoy F, Powers JM. Effects of staining and bleaching on color change of dental composite resins. J. Prosthet. Dent. 200;95(2):137–142. doi: 10.1016/j.prosdent.2005.11.019. 10.1016/j.prosdent.2005.11.019 16473088

[24] Satou N., Khan A.M., Matsumae I., Satou J., Shintani H. (1989 Nov 1). In vitro color change of composite-based resins. Dent. Mater..

[25] Abu-Bakr N., Han L., Okamoto A. (2000). Color stability of compomer after immersion in various media. J. Esthetic Dent..

[26] Cevik P. (2023). Coloring effects of disinfectants on pure or nano-TiO2-incorporated maxillofacial silicone prostheses. Materials.

[27] Eleni P.N., Krokida M.K., Polyzois G.L. (2011). Effects of storage in simulated skin secretions on mechanical behavior and color of polydimethylsiloxanes elastomers. J. Craniofac. Surg..

[28] Polyzois G.L. (1999). Color stability of facial silicone prosthetic polymers after outdoor weathering. J. Prosthet. Dent.

[29] Polyzois G.L., Tarantili P.A., Eng C., Mary J., Andreopoulos A.G., Eng C. (2000). Skin secretions. J. Prosthet. Dent.

[30] Goiato MC, Haddad MF, Pesqueira AA, Moreno A, dos Santos DM, Bannwart LC. Effect of chemical disinfection and accelerated aging on color stability of maxillofacial silicone with opacifiers. J. Prosthodont.. 201;20(7):566–569. 10.1111/j.1532-849X.2011.00755.x 21880094

[31] Gary J.J., Huget E.F., Powell L.D. (2001). Accelerated color change in a maxillofacial elastomer with and without pigmentation. J. Prosthet. Dent.

[32] dos Santos D.M., Goiato M.C., Sinhoreti M.A.C., Fernandes A.Ú.R., Ribeiro P., do P., Dekon SF. de C. (2010). Color stability of polymers for facial prosthesis. J. Craniofac. Surg..

[33] Griniari P., Polyzois G., Papadopoulos T. (2015). Color and structural changes of a maxillofacial elastomer: the effects of accelerated photoaging, disinfection and type of pigments. J. Appl. Biomater. Funct. Mater..

